# Domestic violence against married women during the COVID-19 pandemic in Egypt

**DOI:** 10.1186/s12905-022-01674-5

**Published:** 2022-03-27

**Authors:** Mira M. Abu-Elenin, Ahmed A. Elshora, Mohamed Saad Sadaka, Doaa E. Abdeldaim

**Affiliations:** Faculty of Medicine, Tanta University, Tanta, Egypt

**Keywords:** Violence, Women, COVID-19, Egypt

## Abstract

**Background:**

In face of the COVID-19 pandemic, many countries including Egypt implemented stay indoor rules. These regulations slowed the propagation of the coronavirus, meanwhile they contributed to increase mental health issues, particularly the risk of experiencing intimate partner violence (IPV). That might lead to adverse health and social outcomes on the abused women and the children. This study aimed to examine the effect of the COVID-19 pandemic on the incidence of intimate partner violence against married women in Egypt.

**Methods:**

A cross-sectional study enrolled 2068 married women through an electronic survey link. An anonymous self-administered questionnaire was used. It included demographic data and assessed the frequency of exposure to various forms of spousal violence before and after the COVID-19 pandemic.

**Results:**

The mean age of respondents was 33.8 ± 6.3 years. The commonest types of violent behaviors that have been increased significantly after the COVID-19 pandemic were: twisting arms/pulling the hair (pre 32.8%, post 75%), leaving the house without informing or giving the wife money (pre 12.2%, post 30.3%), restricting interaction with her family members (pre 26.1, post 40.4%), treating her as a servant (pre 28.7%, post 36.7%) and insulting her in front of others (pre 22.9%, post 30.8%).The associated determinants for higher violence rate were; low women education, young age at marriage, low educational and job rank of husband, husband’s tobacco use and reduced family income (p < 0.05).

**Conclusions:**

The overall prevalence of economic and some types of physical and emotionally abusive behaviors have been increased after the emergence of COVID-19 pandemic. Special intervention should be designed to address this issue in collaboration with public health organizations.

## Introduction

A novel viral disease named COVID-19 emerged in Wuhan, China at the end of 2019. Within three months, the epidemic became a global pandemic, that forced governments all over the world to declare social distancing requirements and quarantines [1].

“Stay at home” has become the attitude of public health organizations and governments. But for victims of domestic violence, home is a dangerous place and isolates them from networks of social support. Policies of containment may lead to higher rates of mental distress including domestic violence, substance abuse, depression, anxiety, and suicide. Besides, the resulted job losses and financial instability may aggravate these consequences [2].

Violence against women is defined according to the United Nations as “any act of gender-based violence that leads to physical, sexual or mental harm or suffering to women, including threats of such acts, coercion or arbitrary deprivation of liberty, whether occurring in public or private life.” While, intimate partner violence (IPV) is a physical, psychological, or sexually abusive behavior that inflicts harm on intimate [3].

In 2016, the World Health Organization (WHO) reported that domestic violence is a critical problem worldwide. Global surveys showed that at least 35% of women have experienced physical and/or sexual violence by an intimate partner. The prevalence estimates of intimate partner violence range from 23.2% in high-income countries and 24.6% in the WHO Western Pacific region to 37% in the WHO Eastern Mediterranean region, and 37.7% in the WHO South-East Asia region [4].

Before the COVID-19 pandemic, domestic violence was considered a critical public health issue globally, particularly in the form of intimate partner violence (IPV). Worldwide, 30% of women experience some forms of physical or sexual violence by an intimate partner in their lifetime. It is usually experienced by women and maybe by men [5]. Violence against women is considered a major violation of human rights which has reverse consequences on exposed women’s physical and mental wellbeing as well their children [6].

Data aborted from the Economic Cost of Gender-Based Violence Survey conducted in 2015 by UNFPA and the National Council for Women in Egypt (NCW) showed that 7.8 million women experience all forms of violence annually [6]. Incidence of Domestic violence according to the Egyptian Demographic Health Survey 2014, about one-third of ever-married women aged 15 to 49 years have suffered from violence and physical violence was the most frequent form (36%) [7].

During COVID-19 pandemic, there have been reports from various countries indicating an increase in violence against women. Jianli County Police Station in China (Central Hubei Province) announced that number of intimate partner violence cases in February 2020 was tripled compared to February 2019. In Australia, a survey of 400 frontline workers revealed a 40% increase in “pleas for help” and a 70% increase in case complexity. In Italy, there is a national network of shelters for women who have been subjected to gender-based violence. reported that from 2 March to 5 April 2020, 80 shelters were contacted by 2867, representing a 74.5% increase over the usual monthly records of 2018 [8].

Organizations must quantify the burden of domestic violence on the COVID‐19 pandemic and mobilize resources to address it. Additionally, COVID‐19 testing sites must be partnered with domestic violence response organizations to incorporate screenings for such misbehaviors [9].

It is also, recommended to improve primary prevention by recognizing causes and determinants of violence against women including early marriage, poor education, low economic conditions, and alcohol or substance abuse. It’s crucial to implement intervention programs to reduce this act, taking into consideration the public view of domestic violence as a family matter [10]. Moreover, it is a salient issue which cannot be avoided through personal or community efforts. Therefore, legislation and reform by laws are required to support the women exposed to any kind domestic violence through financial assist, housing and ad-vocal help and social rehabilitation for severely imposed women [11]. Through identifying the dynamics of an abusive relationship and the associated mediators with IPV, we could identify how the COVID-19 can exaggerate those mediators resulting in an increase in abusive behavior and even new kinds of violence [12].

We thought out that identifying the underlying determinants of IPV imposed on married women during the COVID-19 crisis, would enable the concerned authorities to tailor individual and community-based intervention to curb this health issue. During the COVID-19 crisis in Egypt, there was insufficient solid data except for several reports on the media about some cases experienced IPV. Therefore, this study aimed to investigate the effect of the COVID-19 Pandemic on the incidence of intimate partner violence (IPV) against married women in Egypt and the associated predisposing factors.

## Subjects and methods

### Study design and settings

A cross-sectional design was employed for the present study over a period of 3 months during August to October 2020, and it was electronically conducted.

### Target population and sampling

The target population was Egyptian married women aged > 18 years, reside inside Egypt or abroad. Married women aged ≤ 18 years, recently married participants, or whose duration of the marriage is less than 6 months were excluded from the study.

Participants were recruited using a convenience sampling technique, reached through authors' networks and social media platforms due to the current situation of imposed physical distancing as a result of the COVID-19 pandemic.

According to the Egyptian Census 2017 [13], the number of married females above the age of 18 years was 31,500,000 the sample size was computed using the EPI-info 2002 CDC software program with consideration to the following: the power of the study 80%, confidence level at 95% and an error margin of 0.05 and about 33% is the prevalence rate of domestic violence between married Egyptian women according to previous literature [7]; a minimum sample of 1275 married women is required. The final sample size reached 2068 women.

The study participants were recruited using the snowball nonrandom sampling technique. The link to the survey was sent by the authors to many various websites and social networks. The respondent women were kindly asked to share the survey link with their contacts. Therefore, the questionnaire was rolled out with many participants rather than the initial recipients.

A pilot study was performed included 20 married women to make sure of the clarity and reasonability of the measuring tool. The respondents' feedback was taken into consideration.

### Study tools

An electronic survey link was developed using Google survey forms. This tool was chosen due to the social distancing circumstances, and we found it more helpful to collect such kind of critical data remotely since women might be shy or afraid to speak out.

Data was collected by using an anonymous self-administered electronic questionnaire which was initially structured in English, then it has been translated into the Arabic language. Finally, it has been extensively revised by an Arabic language expert before beginning. The questionnaire consisted of three sections, the first included demographic characteristics, marriage history, and history of exposure or diagnosis of COVID-19.

The second part is the Domestic Violence Questionnaire [14] to assess the subjective exposure to domestic violence. It consists of 20 items with dichotomous responses scored “1” for Yes response and “0” for No response.

This self-administered questionnaire is a discriminative instrument that captures the major dimensions of the concept of domestic violence; physical, verbal, psychological ad sexual violence. The overall score was summed up for each respondent, the cutoff score of 5 indicated experiencing domestic violence. The participants were asked to fulfill this questionnaire on one basis of points time: before and after the emergence of the COVID-19 Pandemic.

The third part consisted of 6 questions related to the effect of experienced domestic violence upon women’s maternal and professional roles with a Likert scale from 0 (no effect) to 5 (significant effect) and their knowledge about hot numbers for informing domestic violence events.

### Statistical analysis

The organization, tabulation, presentation, and analysis of data were performed by using SPSS IBM Chicago, version 23. The outcome variables will be examined using the appropriate pre and post-test (Paired t-test). Explanatory variables included demographics, marriage history, and effects of COVID-19 related data. Each explanatory variable was divided into categories and will be presented as frequency number and percentage. The association between the outcome variables and explanatory variables was tested using the Chi-squared test. When the chi-squared test was not appropriate due to the presence of more than 20% of cells with an expected number < 5, the Exact test was be applied instead. The level of significance adopted was p < 0.05.

## Results

Table 1 shows the sociodemographic data of the studied women. The majority of them (62.2%) were between the ages of 31 and 40, and 68.1% lived in Egypt. More than three quarters (81.9%) were from Lower Egypt, while 69.7% were from urban areas. As regards women’s education and employment, 39.9% received postgraduate education and 48.4% worked in governmental jobs. The mean age of women at marriage was 24 years old. As regards marriage length, the women who participated were equally distributed in two groups (37.8% in 6–10 years) and (37.2% in 10–15 years). The proportion of women with two children was the same as the proportion of women with three children (33%, 33.5% respectively). 35.1% of participants reported having a family income that was sufficient for essentials, luxuries, and savings. The basic characteristics of respondents’ husbands are shown in Table 2; their mean age at the time of the study was 38.9 years, while it was 28.5 years at the time of marriage was 38.9 years. About 42% of them had a university education and 39.4% had a governmental job. Nearly half of them (47.3%) were tobacco users, while only 11.7% were alcohol and drug users. According to family income, 57.4% of respondents reported that their income had decreased as a result of the COVID-19 pandemic.

**Table 1:** Characteristics of women in the studied group (n = 2068)

Characteristics	Mean ± SD	Frequency (%)
Age of studied sample	33.8 ± 6.3	
20–30 year	27.1 ± 1.6	605 (29.3%)
31–40 year	35.2 ± 2.5	1287 (62.2%)
41–50 year	42.5 ± 1.5	132 (6.4%)
> 51 year	60.8 ± 5.7	44 (2.1%)
Residence		
Egypt		1408 (68.1%)
Abroad		660 (31.9%)
Residence in Egypt		
Upper Egypt		220 (10.6%)
Lower Egypt		1694 (81.9%)
Suez canal		154 (7.4%)
Geographical region		
Urban		1441 (69.7%)
Rural		627 (30.3%)
Women’s education		
Primary and preparatory		132 (6.4%)
Secondary		407 (19.7%)
University		704 (34%)
Postgraduate		825 (39.9%)
Women’s job		
Housewife		627 (30.3%)
Worker		231 (11.2%)
Free business		88 (4.3%)
Governmental work		1001 (48.4%)
Private wok		121 (5.9%)
Women’s age at marriage	24 ± 2.6	
Length of marriage		
< 1 year		11 (0.5%)
1–5 year		319 (15.4%)
6–10 year		781 (37.8%)
10–15 year		770 (37.2%)
> 15 year		187 (9%)
Number of children		
0		165 (8%)
1		275 (13.3%)
2		682 (33%)
3		693 (33.5%)
4		242 (11.7%)
5		11 (0.5%)
Family income		
Not enough		374 (18.1%)
Essentials		407 (19.7%)
Essentials and luxuries		561 (27.1%)
Essentials, luxuries and save		726 (35.1%)
Women was COVID-19 positive		363 (17.6%)

**Table 2:** Characteristics of women’ husband

Characteristics	Mean ± SD	Frequency (%)
Husband’s age now	38.9 ± 6.9	
Husband’s age at marriage	28.5 ± 2.6	
Husband’s education		
Primary and preparatory		110 (5.3%)
Secondary		440 (21.3%)
University		869 (42%)
Postgraduate		649 (31.4%)
Husband’s job		
No work		22 (1.1%)
Worker		528 (25.5%)
Free business		220 (10.6%)
Governmental work		814 (39.4%)
Private wok		484 (23.4%)
Husband’s tobacco use		979 (47.3%)
Husband’s alcohol or drug use		242 (11.7%)
Husband was COVID-19 positive case		374 (18.1%)
Family income affected by corona		
Not affected		880 (42.6%)
Decreased		1188 (57.4%)
Increased		0 (0%)

Table 3 compares the rate of violence experienced by the studied women before and after COVID-19 respectively. The most frequently reported types of physical violence were slapping at (32.4%, 31.9%), beating at (44.1%, 33.5%), twisting arms/pulling hair at (32.8%, 75%), kicking at (14.4%, 16.5%) and chocking/inflicting burn at (5.3%, 3.2%). The most common types of economic violence were control over handling money by spouses at (33%, 39%), buying stuff at (33.4%, 42.8%) and leaving the house without giving her money at (12.2%, 30.3%). The most reported types of emotional violence were preventing the wife from meeting her female friends at (36.5%, 40%), limiting interaction with family members at (26.1, 40.4%) and treating her as a servant at (28.7%, 36.7%). The most frequent types of verbal violence were insulting in front of others at (22.9%, 30.8%) and threatening to harm her physically at (25.5%, 26.2%). The most frequent types of sexual violence were ignoring sexual relationships for weeks at (34.6%, 35.6%) and forcibly having sexual relationships at (42.1%, 41.2%).

**Table 3:** Description of violence experienced by the studied women

Violence variables	Before COVID-19		After COVID-19		P value
	Yes	No	Yes	No	
1-Did not permit to meet/interact with female friends	755 (36.5%)	1313 (63.5%)	827 (40%)	1241 (60%)	0.02
2-Restricted interaction with your family members	539 (26.1%)	1529 (73.9%)	836 (40.4%)	1232 (59.5%)	0.000*
3- Did not permit to handle money	682 (33%)	1386 (67%)	807 (39%)	1261 (60.9%)	0.000*
4- Did not permit to choose/buy things	691 (33.4%)	1377 (66.6%)	886 (42.8%)	1182 (57.1%)	0.000*
5- Irritated/suspicious/angry if you talked to other men	682 (33%)	1386 (67%)	396 (19.1%)	1672 (80.8%)	0.0001*
6- Accused you of being unfaithful	66 (3.2%)	2002 (96.8%)	55 (2.7%)	2013 (97.3%)	0.3
7- Treated you like a servant	594 (28.7%)	1474 (71.2%)	759 (36.7%)	1309 (63.3%)	0.0001*
8- Did not allow you to partake in decision-making	759 (36.7%)	1309 (63.3%)	649 (31.4%)	1419 (68.6%)	0.0001*
9- He kept away from home for days or weeks without informing you/giving you money	253 (12.2%)	1815 (87.7%)	627 (30.3%)	1441 (69.6%)	0.0001*
10- He was unfaithful to you/had extra-marital relationships	429 (20.7%)	1639 (79.2%)	341 (16.5%)	1727 (83.5%)	0.0001*
11- Did not react against his relatives/agreed with his relatives, when they insulted you	825 (39.9%)	1243 (60.1%)	660 (31.9%)	1408 (68.1%)	0.0001*
12- Insulted you in front of others	473 (22.9%)	1595 (77.1%)	638 (30.8%)	1430 (69.1%)	0.0001*
13- Threatened to harm you physically	528 (25.5%)	1540 (74.4%)	542 (26.2%)	1526 (73.8%)	0.6
14- Slapped you	671 (32.4%)	1397 (67.5%)	660 (31.9%)	1408 (68.1%)	0.7
15- Beat you on other body parts	913 (44.1%)	1155 (55.8%)	693 (33.5%)	1375 (66.5%)	0.0001*
16- Twisted your arm/pulled your hair	678 (32.8%)	1390 (67.2%)	1551 (75%)	517 (25%)	0.0001*
17- Kicked you/dragged you	297 (14.4%)	1771 (85.6%)	342 (16.5%)	1726 (83.5%)	0.05*
18- Choked you or inflicted burns on you	110 (5.3%)	1958 (94.7%)	66 (3.2%)	2002 (96.8%)	0.001*
19- Ignored you purposely, by not having sexual intercourse with you for weeks	715 (34.6%)	1353 (65.4%)	737 (35.6%)	1331 (64.4%)	0.5
20- Had sexual intercourse with you forcibly, when you were not interested	871 (42.1%)	1196 (57.8%)	851 (41.2%)	1217 (58.8%)	0.6

Table 4 shows the relationship between sociodemographic data of studied females and the total violence rate before and after COVID-19. Women aged 31–40 years (55.4%, 51.9%), with low and middle educational levels (38.1%, 43.8%), living in rural areas (59.2%, 61.1%), marrying at young ages of 19 to25 years (89.9%, 92.6%), increasing the length of the marriage 10–15 years (40.4%, 40.2%) and having low family income (34.4%, 39.8%) experienced moderate to high rates of violence more than others during a pre-and post-COVID-19 crisis. Also, females whose husbands were aged 29–34 years (66.5%, 68.5%), had a middle level of education (49.2%, 53.9%), being workers (51.6%, 57.7%) and tobacco users (65.3%, 73.1%) had a higher risk of moderate to high rate of violence.

**Table 4:** Relation between sociodemographic and total violence before and after COVID-19

Sociodemographic data	Total exposure to violence					
	Pre			post		
	Minimal (n = 1173)	Moderate to high (n = 895)	p	Minimal (n = 1252)	Moderate high (n = 815)	p
Age groups of women						
20–30 y	251 (21.4%)	354 (39.6%)	0.0001*	252 (20.1%)	352 (43.2%)	0.0001*
31–40 y	791 (67.4%)	496 (55.4%)		864 (69%)	423 (51.9%)	
41–50 year	110 (9.4%)	22 (2.5%)		118 (9.4%)	14 (1.7%)	
> 51 year	21 (1.8%)	23 (2.6%)		18 (1.4%)	26 (3.2%)	
Residence						
In Egypt	943 (80.4%)	465 (52%)	0.0001*	999 (79.8%)	409 (50.2%)	0.0001*
Abroad	230 (19.6%)	430 (48%)		253 (20.2%)	406 (49.8%)	
Geographical region						
Town	1076 (91.7%)	365 (40.8%)	0.0001*	1124 (89.8%)	317 (38.9%)	0.0001*
Village	97 (8.3%)	530 (59.2%)		128 (10.2%)	498 (61.1%)	
Women’s education						
Primary and preparatory	0 (0%)	132 (14.7%)	0.0001*	0 (0%)	132 (16.2%)	0.0001*
Secondary	66 (5.6%)	341 (38.1%)		49 (3.9%)	357 (43.8%)	
University	503 (42.9%)	201 (22.5%)		510 (40.7%)	194 (23.8%)	
Postgraduate	604 (51.5%)	221 (24.7%)		693 (55.4%)	132 (16.2%)	
Women’s job						
Housewife	229 (19.5%)	398 (44.5%)	0.0001*	227 (18.1%)	399 (49%)	0.0001*
Worker	99 (8.4%)	132 (14.7%)		82 (6.5%)	149 (18.3%)	
Free business	77 (6.6%)	11 (1.2%)		66 (5.3%)	22 (2.7%)	
Governmental work	691 (58.9%)	310 (34.6%)		778 (62.1%)	223 (27.4%)	
Private wok	77 (6.6%)	44 (4.9%)		99 (7.9%)	22 (2.7%)	
Women’s age at marriage						
19–25 year	783 (66.9%)	805 (89.9%)	0.0001*	832 (66.6%)	755 (92.6%)	0.0001*
26–33 year	377 (32.2%)	90 (10.1%)		407 (32.6%)	60 (7.4%)	
> 33 year	11 (0.9%)	0 (0%)		11 (0.9%)	0 (0%)	
Length of marriage						
< 1 year	11 (0.9%)	0 (0%)	0.0001*	11 (0.9%)	0 (0%)	0.0001*
1–5 year	185 (15.8%)	134 (15%)		197 (15.7%)	122 (15%)	
6–10 year	428 (36.5%)	353 (39.4%)		456 (36.4%)	324 (39.8%)	
10–15 year	407 (34.7%)	362 (40.4%)		441 (35.2%)	328 (40.2%)	
> 15 year	142 (12.1%)	46 (5.1%)		147 (11.7%)	41 (5%)	
Husband’s age at marriage						
23–28 year	668 (57.4%)	278 (31.1%)	0.0001*	700 (56.4%)	246 (30.2%)	0.0001*
29–34 year	473 (40.7%)	595 (66.5%)		509 (41%)	558 (68.5%)	
> 35 year	22 (1.9%)	22 (2.5%)		33 (2.7%)	11 (1.3%)	
Children number						
No children	33 (2.8%)	132 (14.7%)	0.0001*	44 (3.5%)	121 (14.8%)	0.0001*
1–3 child	1064 (90.7%)	586 (65.5%)		1134 (90.6%)	515 (63.2%)	
> 4child	76 (6.5%)	177 (19.8%)		74 (5.9%)	179 (22%)	
Husband’s education						
Primary and preparatory	66 (5.6%)	44 (4.9%)	0.0001*	49 (3.9%)	61 (7.5%)	0.0001*
Secondary	0 (0%)	440 (49.2%)		0 (0%)	439 (53.9%)	
University	602 (51.3%)	267 (29.8%)		615 (49.1%)	254 (31.2%)	
Postgraduate	505 (43.1%)	144 (16.1%)		588 (47%)	61 (7.5%)	
Husband’s job						
No work	0 (0%)	22 (2.5%)	0.0001*	7 (0.6%)	15 (1.8%)	0.0001*
Worker	66 (5.6%)	462 (51.6%)		57 (4.6%)	470 (57.7%)	
Free business	121 (10.3%)	99 (11.1%)		126 (10.1%)	94 (11.5%)	
Governmental work	559 (47.7%)	255 (28.5%)		614 (49%)	200 (24.5%)	
Private wok	427 (36.4%)	57 (6.4%)		448 (35.8%)	36 (4.4%)	
Tobacco use						
Yes	395 (33.7%)	584 (65.3%)	0.0001*	382 (30.5%)	596 (73.1%)	0.0001*
No	778 (66.3%)	311 (34.7%)		870 (69.5%)	219 (26.9%)	
Alcohol or drug use						
Yes	77 (6.6%)	165 (18.4%)	0.0001*	60 (4.8%)	182 (22.3%)	0.0001*
No	1096 (93.4%)	730 (81.6%)		1192 (95.2%)	633 (77.7%)	
Family income						
Not enough	66 (5.6%)	308 (34.4%)	0.0001*	49 (3.9%)	324 (39.8%)	0.0001*
Essentials	163 (13.9%)	244 (27.3%)		158 (12.6%)	249 (30.6%)	
Essentials and luxuries	483 (41.2%)	78 (8.7%)		500 (39.9%)	61 (7.5%)	
Essentials, luxuries and save	461 (39.3%)	265 (29.6%)		545 (43.5%)	181 (22.2%)	
Family income affected by corona						
Not affected	462 (39.4%)	418 (46.7%)	0.0001*	508 (40.6%)	372 (45.6%)	0.02*
Decreased	711 (60.6%)	477 (53.3%)		744 (59.4%)	443 (54.4%)	

## Discussion

To reduce the spread of the coronavirus, many countries have implemented strict indoor quarantine. These policies exacerbate the mental and physical health problems of people who are confined in their homes [15]. Along with the COVID-19 pandemic, the home was turned into a dangerous place for victims of intimate partner violence, because they had to spend prolonged hours with their partners and detached from people who support them [16].

This cross-sectional study enrolled 2068 married women all over Egypt. Their mean age was 33.8 ± 6.3 years, ranged from 20 to 64 years. Most of them were residing in Egypt during the time of the study (68.1%) and were of urban residence (69.7%). More than half of participants attained a higher educational level and the same was observed among their husbands. About half of the respondent women worked in the governmental sector (48.4%), compared to (39.4%) of their husbands. Respondents’ mean age at the time of marriage, was younger than their husbands (24 ± 2.6 versus 28.5 ± 2.6 years).

In the current study, the respondents were surveyed regarding the different types of spousal violence; they were experiencing pre- and post-the COVID-19 crisis. Also, the study identified the possible contributors to these violent behaviors.

This study revealed that the overall exposure rate of moderate/high degree of violence was 43.2% before the crisis, which was decreased to 39.2% after the crisis. Whereas the minimal degree of violence had increased from 56.7% to 60.5% after the crisis. As the WHO reported that 30% of women globally experience intimate partner physical or sexual violence during their lifetime and such figures could increase during emergency human crises, and natural disasters [17].

Interestingly, a much more exposure rate of spousal violence was reported in previous Egyptian studies (77%) [18] and 58.1% [10]. This is due to limited access in social services during the lockdown. Similar results were found in Iran (62%) [19], Turkey (67.7%) [20], and 100% in Saudi Arabia [21].

However, there is remarkable variability in the IPV prevalence rate between countries. The multi-country study by the WHO revealed that the lifetime prevalence ranged from 15% in Japan to 71% in Ethiopia and the average rate was 47% [22].

During COVID-19 pandemic, most of the world’s population had to stay at home. So, the situation for women who suffer from abusive relationships getting worse [23]. That was detected in the current study in special types of violent behaviors. Regarding physical insult, the participants considerably suffered from increased exposure particularly twisted arm/pulled hair in about 33% pre compared to 75% post). This puts them at risk for injuries as the incidence rate for fractures among IPV victims is hand 6%, wrist, and forearms 3.4% [15].

Moreover, the verbal insult has been increased from 23% to 30.8%. As regards emotional abuse, 28.7% complained about being treated by their husbands as if they were servants, that has been increased to 36.7% after the crisis. Likely was the emotional neglect in the form of prolonged keeping away from home increased from 12.2% to 30.3% after the pandemic.

It is noted that the autonomy of handling money or buying things was limited among 33% of respondents which increased to 39% and 42.8% respectively after the crisis.

It has been reported that perpetrators of intimate partner abuse, may also limit access to money or health-related such as soap, medications, even accessibility to health services [24]. That explains the increased proportion of women who experienced their husbands' restrictions to handle money from 33% pre-crisis and 39% post-crisis).

On the other hand, some abusive behaviors have been declined significantly as a result of the restricted measures to keep indoors, such as being angry when the woman talks to strange men, or her husband might have extramarital relationships. That could be contributed to adherence to the imposed home containments. Meanwhile, the rate of sexual violence in form of “abundance from the relationship” or “forcible sex” has not changed before and after the pandemic. Though, a considerable proportion of respondents suffered from this type of violence as shown in Table 2.

As half of the world’s population were asked to stay at home to slow down the COVID-19 propagation [25]. The present study detected a declined rate of husbands who used to have extramarital relationships (before crisis 20.7%, after-crisis 16.5%). As well as the decreased number of respondents suffered from neutral husbands' reactions when she was insulted by his relatives (before-crisis 39.9%, after-crisis 31.9%). This decrease is due to confinement measures and indoor restrictions.

In China, the police department reported a tripling of domestic violence cases in February 2020 comparable to February 2019, an estimate of 90% were related to the COVID-19 epidemic [26]. Meanwhile, in the UK, a report noted that deaths from domestic abuse between 23 March and 12 April had more than doubled to 16 deaths compared with the average rate in the previous 10 years [27].

Although data are scarce, media coverage and reports from organizations that respond to violence against women reveal an alarming picture of increased figures of domestic violence during this pandemic [28].

The analysis of basic demographic data about the rate of violence exposure revealed that women aged between 31 and 40 years were at higher risk to violence rate before and after the crisis, incomparable to other age groups. In contrast, a study reported more violence scores among older age groups (45–59 years) [29].

Previous studies indicated that early married women experienced intimate partner violence more than women who married in adulthood; especially those who refrained from education and social insurance due to early marriage [29, 30]. Likely, women in our study who get married at young ages (19–25 years) were exposed to violence more than the others (89.9%).

The present study has concluded that, with the increasing length of the marriage, the rate of lifetime exposure to domestic violence increase as well, this observation was supported by a similar study in Turkey [29].

After computing the total violence scores, our study revealed that women whose husbands aged between 29 and 34 years, were exposed to moderate/high violence rate more than the younger age group. It is thought that the rate of committing lifetime violence increases among men as their ages and durations of marriage increase. Increased marriage length raises women awareness leading them to report their cumulative experience with IPV over their lifetime. Inconsistence to a previous study, which showed that the husband's age at marriage, had no association with the risk of spousal violence [18]. One possible explanation for the contradictory findings is cultural- and area differences between study samples because IPV reporting is highly dependent on the cultural acceptability of IPV, which varies by community and region.

Generally, young age seems to be related to a higher risk of victimization and perpetration of IPV, with age indirectly associated with aggression towards a partner [31, 32]

Of note that, females attained low and middle educational level were exposed to higher intensity of violence, which has been increased during COVID-19 pandemic (14.7%, 38.1%) versus (16.2%, 43.8%). The great majority of research referred to low levels of education as a predisposing factor for abusing and victimization [32]. However, in some societies with a male-dominant culture, women who received high education, can put them at risk for IPV. This note has been linked with the tendency of highly educated women to confront their partner autonomy [33].

As regards, the educational level of husbands, those who attained the middle level of education committed a higher rate of violence (49.2%) against their wives than their counterparts. This rate has been increased to 53.9% after the crisis. Women whose husbands involved in manual work or inferior jobs represented about 25%. Those were at greater risk of experiencing moderate to high spousal violence than other jobs. Since it is well known that skilled jobs have been declined and manual workers largely suffered during the lock down era of COVID. This rate has been increased from 51.6% to 57.7% after the crisis. These findings were parallel to previous studies [18, 34, 35]

Increased rates of intimate partner violence has been reported during times of crisis, as a result of economic insecurity and stressful environments [36]. Previous findings were parallel to the observed increased violence rate in families whose income was decreased because of the COVID-19 pandemic. It has been documented that, unemployment and men of lower socioeconomic status have a higher risk for committing abuse and tend to inflict more severe forms of violence than their counterparts [37, 38].

It is worthy to mention that the rate of violence exposure among participants, who lived abroad, was less than those who lived inside Egypt. Of note that, women who inhabited rural areas were at increased risk for moderate/high degree of violence (59.2%) which has been exacerbated during the crisis to (61.1%). That agrees with previous studies [18, 39]. Though, Ali et al. 2017, detected no statistically significant association between DV and woman’s residence.

The family income has been decreased in 57.45% of respondents. This could be explained through the disturbed livelihoods during the humanitarian crisis. Also, the inability to earn a living with declined accessibility to basic needs and services could aggravate the existed stress [23]. The household tension could initiate or exaggerate domestic violence. As people stay indoors, families spend a prolonged duration in close contact, including in distressed conditions [40]. Therefore, the rate of moderate/high violence exposure was more observed among women with decreased family income. This finding became more evident after the crisis as shown in Table 4.

A higher proportion of women reported suffering from moderate/high spousal violence if their husbands were tobacco users before and after the crisis (65.3%, 73.1% respectively). And the same was noticed regarding alcohol consumption (18.4%, 22.3%). Similar findings were replicated in other related studies [10, 18]. Moreover, high rates of smoking and substance use disorders were found among maritally violent men. As a result, domestic violence intervention programs should routinely screen for smoking and advise participants to try to quit smoking [41].

The detachment from social and protective networks may further exaggerate the perpetrator's violence. As well, family and friends provide psychological and social support against intimate partner violence [23]. The proportion of respondents who suffered from restricted interaction with their female friends or family members have been increased from (36.5%, 26.1% respectively) before the pandemic to (40%, 40.4% respectively) after the COVID-19 pandemic. This kind of abuse is known to be increased during public health crises, including conflict and natural disasters [42]. In addition, such observation might be attributed to the already implemented social distancing and quarantine measures.

Conclusively, the challenges caused by the COVID-19 pandemic, including stress management of the disease, the reformulation of the household routine, spending more time with the partner isolation from other people outside the house, and financial burden can considerably induce /or increase distress in a previously tensed relationship and predispose domestic violence [43].

## Conclusions

This study identified several sociodemographic factors that might contribute to increase the likelihood of women’s exposure to intimate partner/spousal abuse. He imposed physical and emotional violent behavior has been increased during the COVID-19 pandemic. The low educational level of participated women and their husbands, young age at the time of marriage poor employment status, low income, and residence were significantly associated with spousal violence. Moreover, the humanitarian crisis, like the COVID-19 pandemic exacerbated those factors in precipitating spousal violence episodes.

### Recommendation

Social policies should be adopted to control the virus propagation meanwhile it should compromise the early detection and protection of women suffering from partners’ abusive behavior. Including access to support and health care services, which should be prepared to provide an adequate response considering the family context. Further data is still needed to explore the implications of the COVID-19 pandemic on predators of IPV and domestic violence.

### Limitation of the study

The cross-sectional design of this study is first limitation since it doesn’t prove causal relationship and results of this work can’t be generalized to the total female population in Egypt. The second limitation was related to reluctance of some invited women to participate in the study due to network issues or they were busy and forgot to participate. Lastly this study recruited only married women but engaged or women in a fair were excluded.

## Data Availability

The datasets generated and/or analyzed during the current study are not publicly available due to the belonging of data to the authors’ affiliated institute (Faculty of Medicine -Tanta University), as well as to assure the privacy of respondents’ information. But data would be available from the corresponding author on reasonable request.

## Abbreviations

COVID-19: Novel corona virus disease
IPV: Intimate partner violence
NCW: The National Council for Women in Egypt
UNFPA: United Nation for Population Fund
WHO: World Health Organization
